# The Role of Chemokines in Obesity and Exercise-Induced Weight Loss

**DOI:** 10.3390/biom14091121

**Published:** 2024-09-04

**Authors:** Wenbi He, Huan Wang, Gaoyuan Yang, Lin Zhu, Xiaoguang Liu

**Affiliations:** 1 Graduate School, Guangzhou Sport University, Guangzhou 510500, China; 105852022100037@stu.gzsport.edu.cn (W.H.); 105852023100044@stu.gzsport.edu.cn (H.W.); 105852022400308@stu.gzsport.edu.cn (G.Y.); 2 School of Sport and Health, Guangzhou Sport University, Guangzhou 510500, China; 3 Guangdong Provincial Key Laboratory of Physical Activity and Health Promotion, Guangzhou Sport University, Guangzhou 510500, China

**Keywords:** exercise, chemokines, obesity, adipose tissue, inflammation response

## Abstract

Obesity is a global health crisis that is closely interrelated to many chronic diseases, such as cardiovascular disease and diabetes. This review provides an in-depth analysis of specific chemokines involved in the development of obesity, including C-C motif chemokine ligand 2 (CCL2), CCL3, CCL5, CCL7, C-X-C motif chemokine ligand 8 (CXCL8), CXCL9, CXCL10, CXCL14, and XCL1 (lymphotactin). These chemokines exacerbate the symptoms of obesity by either promoting the inflammatory response or by influencing metabolic pathways and recruiting immune cells. Additionally, the research highlights the positive effect of exercise on modulating chemokine expression in the obese state. Notably, it explores the potential effects of both aerobic exercises and combined aerobic and resistance training in lowering levels of inflammatory mediators, reducing insulin resistance, and improving metabolic health. These findings suggest new strategies for obesity intervention through the modulation of chemokine levels by exercise, providing fresh perspectives and directions for the treatment of obesity and future research.

## 1. Introduction

Obesity has become a major public health challenge globally, and according to the World Health Organization (WHO), approximately 1.9 billion adults are overweight, with more than 650 million of them being obese. Obesity rates are projected to exceed 18 per cent for men and 21 per cent for women by 2025 [1]. In European countries such as Cyprus and Lithuania, childhood obesity is prominently evidenced by significant rates of overweight or obesity [2,3]. By 2030, it is expected that 45 million children over the age of 5 will be obese in South and Southeast Asia. Meanwhile, China’s obesity rate is also rising to varying degrees among both urban and rural populations, predicting a significant increase in obesity rates among both adults and children [4,5,6]. Obesity is strongly associated with a wide range of chronic diseases, including cardiovascular disease, diabetes, atherosclerosis, dyslipidemia, and hypertension, exacerbating the public health burden. Therefore, effective interventions are urgently needed [7,8,9].

Chemokines are a class of signaling molecules that regulate the aggregation of cells towards sites of inflammation and play a key role in macrophage infiltration of adipose tissue. In adipose tissues, the high expression of chemokines significantly impacts the formation and development of obesity-related conditions. For instance, C-C motif chemokine ligand 2 (CCL2) and C-C motif chemokine ligand 5 (CCL5) are closely associated with obesity-induced cardiovascular disease and insulin resistance [10,11,12]. Research has indicated that genetic knockout or pharmacological inhibition of chemokine pathways have improved glucose tolerance, insulin sensitivity, hyperlipidemia, and steatohepatitis in *mice* [13,14,15]. Moreover, downregulating chemokines and their receptors can decrease the inflammatory responses induced by obesity, prevent the onset of insulin resistance, and facilitate the upregulation of mitochondrial oxidative phosphorylation and fatty acid metabolism pathways, for example, in brown adipocytes as seen with CCL5 [16,17]. In addition, the inhibition of chemokines suppresses the inflammatory response in the liver and adipose tissue, reduces the number of immune cells, decreases circulating cytokine levels, and limits adipogenesis and fatty acid uptake. These effects work together to reduce hepatic fat deposition, improve insulin sensitivity, and help reduce obesity symptoms [18,19]. Thus, the development of regulatory strategies targeting chemokines offers a promising avenue for ameliorating obesity.

For example, running has been observed to attenuate inflammatory responses induced by a high-fat diet (HFD) in *mice* with obesity, subsequently impacting obesity metrics [20]. High-Intensity Interval Training (HIIT) not only significantly increases the expression of C-C chemokine receptor type 5 (CCR5) in immune cells but also decreases levels of inflammatory mediators such as the Interleukin-6 (IL-6) receptor and Monocyte Chemotactic Protein-1 (MCP-1) in plasma and adipose tissue, potentially ameliorating health issues associated with overweight and obesity. Meanwhile, Moderate Intensity Continuous Training (MICT) has been shown to reduce the expression of C-C chemokine receptor type 2 (CCR2) and C-X-C chemokine receptor type 2 (CXCR2), further confirming that the type and intensity of exercise directly regulate immune cell function and inflammatory states [21,22]. In addition, studies have also shown that exercise improves the vascular repair capacity of Endothelial progenitor cells (EPCs) in older adults by enhancing the C-X-C chemokine receptor type 4 (CXCR4)/*Janus kinase-2 (JAK-2)* signaling pathway, revealing a potential mechanism of exercise as a potential mechanism of anti-aging strategy [23].

Vigorous exercise mobilizes specific monocyte subpopulations through a catecholamine-dependent mechanism, in particular a significant increase in the number of CD14^+^CD16^+^ monocytes [24]. Meanwhile, acute exercise-induced changes in cortisol regulate the expression and migratory activity of monocyte CCR2, affecting the inflammatory response and tissue repair [25]. Exercise *activates the transcription factor 3 (ATF3)*, which reduces chemokine and cytokine expression in mouse skeletal muscle; this supports molecular adaptations to endurance training [26]. The aim of this paper is to explore how exercise adjusts chemokine expression to improve the health of obese patients and reveal its potential therapeutic role in weight loss and tissue repair. We also highlight the need for an in-depth exploration of the mechanisms by which exercise affects chemokine-mediated pathways.

## 2. Obesity Overview

**Figure 1:**
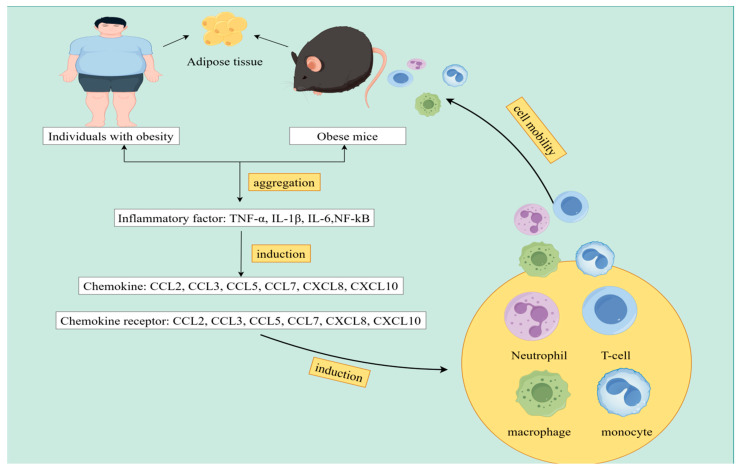
The process of adipose tissue inflammation, chemokine expression, and immune cell migration induced by obesity. By Figdraw (www.figdraw.com, accessed on 20 August 2024). In obese conditions, adipose tissues accumulate large quantities of inflammatory mediators, including TNF-α, IL-1β, IL-6, and NF-kB. These inflammatory factors further induce the expression of various chemokines such as CCL2, CCL3, CCL5, CCL7, CXCL8, and CXCL10. Chemokines bind to specific receptors—including CCR1, CCR2, CCR3, CCR5, CXCR1, CXCR2, and CXCR3—initiating the migration of immune cells towards the adipose tissue, particularly T cells, neutrophils, monocytes, and macrophages. This migration enhances the inflammatory response within obese adipose tissues.

Obesity is defined as a chronic condition with body mass index (BMI) ≥ 30 kg/m^2^, caused by multiple factors such as genetics, environmental factors, poor dietary patterns, sedentary lifestyle, and endocrine disorders [7,27,28,29,30]. Obesity triggers Nonalcoholic Steatohepatitis (NASH). It causes liver fat accumulation, inflammation, and fibrosis through increased serum Lipopolysaccharide (LPS) levels and activation of *toll-like receptor 4 (TLR4)* pathway, which severely impairs liver function and overall health. Obesity also increases the risk of hypertension, dyslipidemia, and other cardiovascular diseases in children in adulthood [31,32]. In addition, obesity not only leads to excessive accumulation of liver fat, but also triggers the risk of cancers, and may lead to a poor cancer prognosis [27]. Based on BMI, obesity can be categorized into three grades: Grade I (30.0–34.9), Grade II (35.0–39.9), and Grade III (40.0 and above) [7], Figure 1.

In terms of metabolic health, people who are obese or overweight have a 1.74 times higher risk of cardiovascular disease compared to those of normal weight. Obesity is strongly associated with high blood pressure. Obesity causes chronic low-grade inflammation of visceral adipose tissue, which increases the risk of cardiovascular and metabolic diseases and affects vascular function, increasing the risk of developing hypertension [33,34].

Moreover, obesity is strongly associated with the risk of developing Obstructive Sleep Apnea Hypopnea Syndrome (OSAHS). Individuals with obesity have a higher likelihood of suffering from this condition, which can potentially lead to insulin resistance and glucose metabolism disorders [35]. Women with central obesity and metabolic syndrome are also at risk of developing sleep apnea OSAHS [36]. Specifically, the degree of obesity is positively associated with the severity of OSAHS, which has important implications for both sleep and breathing [37,38]. Systemic chronic inflammation due to obesity may result in insulin resistance and impaired pancreatic β-cell function, increasing the risk for type 2 diabetes [9].

In addition, obesity is positively associated with the risk of gout and hyperuricemia, with the prevalence of disease increasing with weight gain [39]. Genetic factors and higher BMI significantly increased the risk of gout, with each standard deviation increase in BMI significantly increasing the risk of gout and serum uric acid concentration. The significant association between obesity and gout risk was confirmed even after adjusting for waist-to-hip ratio [40]. Obesity not only puts children at risk of osteoporosis and chronic inflammation, but may also increase the risk of cardiovascular disease, while being associated with metabolic syndrome and its concomitant multiple cardiometabolic risk factors [9,41]. Obesity not only affects an individual’s quality of life, but also significantly increases the risk of developing a wide range of chronic diseases, making the prevention and treatment of obesity an urgent public health challenge.

## 3. Chemokine Overview

Chemokines are a class of small molecule proteins that play a key role in immune responses and regulate inflammatory and immune processes by directing immune cell migration. Regarding the current chemokine nomenclature system, *human* chemokines are mainly used as the standard for categorization and nomenclature [42]. Chemokines are small molecule proteins with molecular weights between 8 and 14 kDa. They play a pivotal role in immune function by regulating the migration of various leukocytes through interactions with G protein-coupled receptors (GPCR) [42,43]. In *humans*, approximately 50 chemokines have been identified [44]. These chemokines are classified into four main families based on their structural features: CXC, CC, XC, and CX3C [42,45,46,47]. Upon binding to their corresponding receptors, chemokines activate intracellular signaling pathways that regulate cell migration, proliferation, survival, and differentiation. Chemokines are essential for the maintenance of immune system development, homeostasis and function by directing immune directed migration. About 20 chemokine receptors have been identified, including C-C chemokine receptor type 1 (CCR1) to C-C chemokine receptor type 9 (CCR9), C-X-C chemokine receptor type 1 (CXCR1) to C-X-C chemokine receptor type 8 (CXCR8), X-C motif chemokine receptor 1 (XCR1), C-X3-C Motif Chemokine Receptor 1 (CX3CR1), which all belong to the GPCR family [42,44,48,49]. The role of chemokines and their receptors in a variety of diseases suggests that they may be potential targets for the treatment of a number of diseases, including obesity (Table 1 and Table 2). 

### 3.1. CXC Chemokines

CXC chemokines are a class of cytokines with specific structural features that play important roles in immune and inflammatory responses. CXC chemokines constitute a subclass of cytokines distinguished by the presence of two cysteine residues at the N-terminus separated by a single amino acid [42,45]. These chemokines are composed of four cysteine residues and can be further subdivided based on the presence or absence of the glutamate-leucine-arginine (ELR) motif adjacent to the first cysteine residue. The ELR-positive CXC chemokines primarily recruit neutrophils, while the ELR-negative chemokines are more chemotactic for lymphocytes [46]. The CXC chemokine family encompasses 17 members: C-X-C motif chemokine ligand 1 (CXCL1) through C-X-C motif chemokine ligand 17 (CXCL17). Among them, C-X-C motif chemokine ligand 8 (CXCL8), which is also known as Interleukin-8 (IL-8), and C-X-C motif chemokine ligand 12 (CXCL12), known as stromal cell-derived factor 1 (SDF-1), are noteworthy [45,47,48,50]. These chemokines play a pivotal role in recruiting neutrophils and T cells to the site of infection or injury. Accordingly, chemokines are critically important in mediating inflammatory responses and regulating immune functions [42,46,50].

In the immune system, CXC chemokines perform essential functions through interactions with receptors ranging from CXCR1 to CXCR8 [42,48]. For instance, CXC chemokines and their receptors are significantly involved in several disease states, including ulcerative colitis, Crohn’s disease, and psoriasis [46]. CXCL9 and CXCL10 interact with the CXCR3 receptor, which can regulate gene expression in hepatic stellate cells and, accordingly, suppress both fibrosis-related genes and collagen expressions, thereby exerting an anti-fibrotic effect. On the other hand, the CXCL12/CXCR4 axis is suggested to be another potential therapeutic target for mitigating inflammation and fibrosis [50]. Additionally, During the development of obesity-related diseases, chemokines such as CXCL5 and CXCL8, secreted by white adipose tissue, are highly expressed in the adipose tissue of obese subjects. These factors not only diminish insulin responsiveness in muscle but also exacerbate insulin resistance [51]. These findings highlight CXC chemokines’ functions in modulating inflammation and immune responses across multiple disease states.

#### 3.1.1. CXCL8

CXCL8, also known as IL-8, binds to the receptors CXCR1 and CXCR2. It is produced by a variety types of cells including Tumor-Associated Macrophages (TAM), adipocytes, fibroblasts, monocytes, epithelial cells, and endothelial cells. CXCL8 is a peptide composed of 72 amino acid residues with a structure consisting of three antiparallel β-folds and an α-helix, stabilized by disulfide and hydrogen bonds, exists in reversible monomeric and dimeric forms, and binds with high affinity to CXCR1 and CXCR2, reinforced by ELR motifs [52,53,54,55,56,57]. CXCL8 is a key chemical mediator that is essential for the initiation and activation of neutrophils, especially during the immune system’s response to infection and tissue damage in the *human* body. CXCL8 regulates leukocyte chemotaxis and activation by interacting with *human* CXC-type chemokine receptors CXCR1 and CXCR2 [54,57]. CXCL8 plays a key role in tumor progression, such as promoting tumor cells proliferation, invasion, and transformation. It has also been implicated in tumor immune escape mechanisms. CXCL8 has the potential to serve as a prognostic biomarker and therapeutic target [56,58].

Moreover, CXCL8 contributes to atherosclerosis pathogenesis through multiple mechanisms, including attracting immune cells to the lesion site, promoting the migration of vascular cells, and contributing to plaque instability [52]. Emerging research indicates that CXCL8 is critical in obesity pathophysiology, driving hepatic inflammation and damage through the chemotaxis of neutrophils and macrophages to the liver [59]. Earlier studies have demonstrated that neutrophils secrete more CXCL8 after being activated by lipopolysaccharide (LPS) and N-Formyl-Met-Leu-Phe (fMLP), highlighting a potential link to the inflammatory response associated with obesity [60]. Whole-genome transcriptome sequencing has identified the CXCL8 gene as playing a role in both individuals with obesity and those with metabolic impairments. This suggests that CXCL8 may serve as an early biomarker for metabolic risk in these populations [61].

#### 3.1.2. CXCL9

CXCL9, also known as Monokine induced by Interferon-γ (MIG), is a low molecular weight protein secreted by cells in response to interferon-γ stimulation and is produced mainly by macrophages and endothelial cells. It activates the receptor CXCR3 through its N-terminal interaction with the N-terminal and extracellular structural domains of the receptor. In this way, chemokine CXCL9 directs the migration of immune cells toward sites of inflammation [62,63,64,65]. CXCL9 induces chemotaxis and participates in immune and inflammatory processes by directing T cells and Natural Killer cells (NK) to migrate to sites of inflammation. CXCL9 plays an important role in tumor immunosurveillance and antitumor immunity [63,64,65,66].

#### 3.1.3. CXCL10

CXCL10, also known as interferon-Gamma-Induced Protein 10 (IP-10) with a molecular weight of approximately 10 kDa. It is secreted by various cell types, including monocytes, endothelial cells, dendritic cells, neutrophils, and astrocytes. This chemokine is characterized by the presence of the CXC motif, where the first two highly conserved cysteine residues are separated by a single non-cysteine amino acid [67,68,69]. CXCL10 binds specifically to its receptor, CXCR3, which is a G-protein-coupled receptor with seven transmembrane domains. After that, CXCL10 can induce the migration of T cells, eosinophils, monocytes, and NK cells toward sites of inflammation, playing a crucial role in both autoimmunity and inflammatory responses [67]. CXCL10 is expressed in islet cells of type 1 diabetic patients but not in the pancreas of healthy subjects. Similarly, in a mouse model of type 1 diabetes, the levels of CXCL10 increase as the disease progresses [68]. In patients with obesity, elevated circulating levels of the CXCL10 chemokine have been observed. These levels correlate with markers of obesity such as BMI, waist circumference, and Homeostatic Model Assessment of Insulin Resistance (HOMA-IR). Elevated CXCL10 may enhance leukocyte adhesion to endothelial cells, contributing to abnormal endothelial function. This process is potentially involved in the development of insulin resistance and obesity-related cardiovascular complications [70]. In addition, CXCL10 gene expression in adipose tissue from obese individuals was found to be significantly correlated with several inflammatory markers, including Interleukin-1 beta (IL-1β), Interleukin-2 (IL-2), IL-6, and CCL2, suggesting that CXCL10 could serve as a potential inflammatory biomarkers [71].

#### 3.1.4. CXCL14

CXCL14, also known as Breast- and Kidney-Expressed Chemokine (BRAK), is mainly expressed in healthy epithelial tissues such as epithelial cells, adipose tissues, intestines, mast cells, and kidneys, and is also detected in tumor cells, fibroblasts, and macrophages. CXCL14, with a molecular weight of approximately 8–12 kDa, contains four cysteine residues forming two disulfide bonds. The main receptors for CXCL14 remain to be conclusively identified [72,73,74]. CXCL14 has a variety of functions, including tumor suppression, antimicrobial, anti-inflammatory, and cell migration regulation [72]. In obese patients, especially those with type 2 diabetes mellitus, CXCL14 levels usually show a decrease in blood circulation and subcutaneous adipose tissue; In addition, there was an inverse correlation between its level and the expression of inflammation-related genes, whereas a positive correlation was observed with insulin sensitivity markers such as Glucose Transporter Type 4 (GLUT4) and lipocalin. These findings imply that downregulation of CXCL14 may serve as a potential biomarker for obesity-related metabolic dysfunction [75].

### 3.2. CC Chemokines

CC chemokines are another important class of chemotactic cytokines that are necessary in regulating the chemotaxis and activation of immune cells. CC chemokines are characterized by two adjacent cysteine residues near the amino terminus. These factors are secreted by different types of cells, including lymphocytes, eosinophils, neutrophils, and monocytes. CC chemokines mediate immune cell chemotaxis and activation by interacting with G protein-coupled receptors on target cells. Activation of these receptors triggers a series of changes via G proteins that activate downstream signaling pathways, such as the phosphatidylinositol signaling pathway, triggering changes in calcium flux and activating protein kinase C [45,46,50,76,77]. The CC chemokine subfamily includes numerous ligands, such as C-C motif chemokine ligand 1 (CCL1) to C-C motif chemokine ligand 28 (CCL28), and each one possesses unique receptor affinities and biological activities [45,47,50,76].

CC chemokines have complex effects in tumors, not only promoting tumor cell proliferation, migration, and invasion, but also recruiting associated cells to participate in immune and inflammatory responses. These factors can express dual functions by regulating anti-apoptosis and drug resistance in cancer cells, which may be both pro- and anti-cancer [76,77]. CC chemokines also attract and activate immune cells such as macrophages, lymphocytes and neutrophils [78]. Ligands for CC chemokines such as CCL2, CCL7, CCL8, CCL11, CCL13 to CCL17, CCL22 to CCL24, and CCL26 can bind to receptors from CCR1 to CCR10 [45,46,47,76,77].

CC chemokines bind to receptors from CCR1 to CCR9 and promote immune cell activation, proliferation, differentiation, and migration to sites of inflammation, thus playing a central role in the regulation of immune responses [42]. The binding of CCL2 to CCR2, especially, activates monocytes, T cells, and basophils, reinforcing their chemotactic and functional activities [45,47]. In addition, CC chemokines interact with G protein-coupled receptors to regulate the migration and activation of leukocytes towards the site of inflammation and modulate the inflammatory response [46]. Altered CC chemokine expression in adipose tissue of patients with obesity may contribute to insulin resistance and diabetes mellitus, suggesting that interventions targeting CC chemokines provide preventive and therapeutic strategies for type 2 diabetes [79]. An in-depth understanding of the mechanism of action of CC chemokines in obesity and its associated complications opens up the possibility of developing new therapeutic strategies.

#### 3.2.1. CCL2

CCL2, also known as MCP-1, is a chemokine produced by a variety of cells including tumor cells, monocytes, macrophages, endothelial cells and other inflammatory cells. This mature monomeric chemokine consists of 76 amino acids and forms a stable tertiary structure through two disulfide bonds. The specific binding of CCL2 to the receptor CCR2 promotes the directed movement of immune cells and is essential for the maintenance of immune surveillance. During tissue injury or infection, CCL2 expression is upregulated to recruit monocytes and other immune cells to sites of inflammation [80,81,82,83,84,85]. CCL2 also regulates the function of various myeloid cells. It has a major impact on a variety of refractory diseases, including various types of cancer, atherosclerosis, multiple sclerosis and metabolic diseases [81,83]. Further studies of CCL2 are essential to elucidate its mechanism of action and physiological significance.

CCL2, as a key immunomodulatory molecule, promotes the migration of immune cells to adipose tissue and has a significant impact on the inflammatory process associated with obesity [10,86]. In the inflammatory response, CCL2 attracts immune cells to adipose and injured tissues, and leads to macrophage infiltration. Furthermore, CCL2 level has been found to decrease with weight loss [10]. The pathway of elevated CCL2 gene expression associated with obesity specifically involves Interferon-gamma (IFN-γ) pretreatment. This treatment leads to acetylation of lysine 27 on histone 3 (H3K27), which amplifies LPS-induced CCL2 expression in monocytes [87]. CCL2 induces inflammation and monocyte recruitment in adipose tissue via its receptor CCR2, which is associated with the exacerbation of metabolic diseases such as atherosclerosis [47]. Previous research has demonstrated that, although CCL2 knockdown via specific genetic or pharmacological approaches does not prevent macrophage infiltration, the absence of CCL2 exacerbates metabolic dysfunctions, such as impaired insulin sensitivity or glucose homeostasis [13].

Furthermore, knocking out the G protein-coupled receptor 21 gene (GPR21) decreased monocyte chemotaxis toward CCL2. This was accompanied by a reduction in CCL2 expression and attenuation of downstream signaling. Consequently, inhibiting GPR21 activity may offer a compelling approach to mitigate obesity-induced insulin resistance and CCR2-mediated inflammatory responses [88]. CCR2 antagonists have potential clinical use in the treatment of inflammatory diseases, pain, and metabolic and cardiovascular diseases [80]. In *mice* subjected to a high-fat diet, elevated CCL2 levels in skeletal muscle are associated with an increase in inflammatory cell infiltration, and enhanced binding to the CCR2 receptor is observed after a high-fat intake [89].

#### 3.2.2. CCL3

CCL3, also known as Macrophage Inflammatory Protein-1 alpha (MIP-1α), is a cytokine belonging to the CC chemokine family. CCL3 is produced by macrophages, B cells, tumor cells, and dendritic cells. The receptors for CCL3 are CCR1 and CCR5 [14,90,91,92,93]. CCL3 interacts with CCR5 and can promote the movement and infiltration of colorectal adenocarcinoma cells. This phenomenon suggests that CCL3 affects colorectal cancer’s progression and metastasis through cancer cell motility [91]. Additionally, CCL3 can enhance the migration of *human* neutrophils, and this effect is particularly broad in neutrophils pretreated with Granulocyte-Macrophage Colony-Stimulating Factor (GM-CSF). The enhanced migration is important for recruiting neutrophils to inflammation sites. These findings highlight the crucial role of CCL3 in inflammatory responses and immune regulation [90]. In the obese state, the expression of CCL3 is elevated in both circulating blood and adipose tissues, playing a significant role in the development of Non-alcoholic fatty liver disease (NAFLD), which is induced by diets high in cholesterol and fat [14,94]. Furthermore, the inhibition of CCL3 has been shown to mitigate liver inflammation, and to improve hepatic insulin resistance and steatosis [14]. This suggests that targeting CCL3 could be a potential therapeutic strategy for managing NAFLD and its associated metabolic disturbances.

#### 3.2.3. CCL5

CCL5, also known as regulated upon activation, normal T cell expressed and secreted (RANTES), is a chemokine predominantly produced by NK cells, T cells, dendritic cells, and macrophages/monocytes. This chemokine interacts with multiple receptors, notably CCR1, CCR3, and CCR5, to recruit and activate immune cells during inflammatory and immune responses. The broad range of cell types capable of producing CCL5, along with its interaction with diverse receptors, underscores its significance in mediating immune and inflammatory responses [16,95,96,97]. CCL5 participates in anti-tumor activity and physiological processes such as cell migration and immune response [16,96]. In the obese state, adipose tissue enhances signaling from CCL5 and its receptor CCR5 to suppress inhibition of AMP-activated protein kinase (AMPK), which decreases lipolysis and oxidative metabolism. This ultimately results in a reduction of energy expenditure and adaptive thermogenesis, further exacerbating the development of obesity and insulin resistance [17]. The expression levels of CCR5 and CCL5 were found to be significantly elevated in adipose tissue during inflammation and insulin resistance associated with obesity. This upregulation plays a crucial role in the recruitment and polarization of adipose tissue macrophages (ATMs), which are pivotal in the development of insulin resistance. Specifically, genetic deletion of CCR5 in a mouse model leads to a reduction in the number of ATMs and shifts ATMs towards the M2 phenotype. These findings underscore the importance of CCR5 in modulating adipose tissue inflammation and suggest its potential as a therapeutic target for ameliorating insulin resistance in obesity [98]. Multiple studies have shown that CCL5 enhances insulin signaling in the hypothalamus through the CCR5 receptor, thereby increasing systemic insulin responsiveness [97].

#### 3.2.4. CCL7

CCL7, also known as Monocyte Chemoattractant Protein-3 (MCP-3), is a single-chain polypeptide consisting of 99 amino acids, including a 23-amino-acid signal peptide and a 76-amino-acid mature form. It is produced by tumor cells, fibroblasts, endothelial cells, vascular smooth muscle cells, and bone marrow mononuclear cells. CCL7 binds to receptors such as CCR1, CCR2, CCR3, and CCR5 [99,100,101]. As a chemoattractant, CCL7 facilitates the migration and accumulation of monocytes and macrophages through its specific receptors and plays a role in various physiological processes, including cell migration, immune response, inflammation, and tumor development [99,100]. CCL7 has a strong affinity for its primary receptor, CCR2 that mediates the pathophysiological mechanisms in monocytes and macrophages. CCL7 effectively recruits leukocytes to sites of inflammation through receptor binding [101]. In murine models of diet-induced obesity and hepatic steatosis, overexpression of CCL7 confers protection against these conditions [102]. A significant role of CCL7 and adiposity has been identified in facilitating the migration and infiltration of inflammatory cells [103]. In the context of obesity and distal sensorimotor polyneuropathy (DSPN), CCL7 serves as a potential inflammatory biomarker, reflecting its correlation with BMI and waist circumference, and appearing to partially mediate the relationship between obesity and DSPN [104].

### 3.3. XC

XC is a member of the C family of chemokines, with its sole ligand being C motif chemokine ligand 1 (XCL1), also known as lymphotactin. This small protein is primarily secreted by activated immune cells, including CD8^+^ T cells, NK cells, mast cells, and Th1-polarized CD4^+^ T cells [105,106,107,108,109]. XCL1 is recognized for its unique ability to bind to the 7-transmembrane G-protein-coupled receptor XCR1. It is distinguished as the only chemokine that forms a disulphide bond, featuring a metastable folding structure. This enables XCL1 to alternate between conventional chemokine folds and unique β-folded dimers [108,110,111,112]. Although the XC chemokine family has fewer members, its role in immunomodulation cannot be ignored.

XCL1 interacts with XCR1 to recruit activated T cells and NK cells [108]. Inhibiting the XCL1-XCR1 interaction may offer therapeutic benefits in treating various inflammatory conditions. However, enhancing this signaling pathway also improves responses to severe infections and cancer treatments [111]. Moreover, XCL1 contributes to bactericidal activities and the immune response [110]. The specific role and systemic regulation of XCL1 in obesity is not fully understood; however, its specific expression in regions of fat deposition suggests a complex link to obesity [112]. Studies of XC chemokines have uncovered new roles for them in obesity and immune responses, providing new perspectives for future therapies.

**Table 1:** Summary of data on chemokines: primary sources, receptors, main effects, and key references.

Chemokine Name	Primary Sources	Acceptor	Primary Role	References
CXCL8 (IL-8)	Tumor-associated macrophages (TAM), adipocytes, fibroblasts, monocytes, Epithelial cells, endothelial cells	CXCR1, CXCR2	CXCL8 is a cytokine that regulates the chemotaxis and activation of leukocytes, such as neutrophils and macrophages. It not only promotes tumor cell proliferation, invasion, and metastasis in tumor development and participates in tumor immune escape, but also causes plaque instability in atherosclerosis by attracting immune cells and promoting vascular cell migration. In liver pathology, CXCL8 drives inflammation and injury.	[52,53,54,55,56,57,58,59]
CXCL9 (MIG)	Macrophages and endothelial cells.	CXCR3	CXCL9 has the ability to induce chemotaxis in T cells and NK cells, directing them to sites of inflammation to participate in immune and inflammatory responses, and plays a role in tumor immunosurveillance and anti-tumor immunity.	[62,63,64,65,66]
CXCL10 (IP-10)	Monocytes, neutrophils, endothelial cells, astrocytes, and dendritic cells.	CXCR3	CXCL10 directs the migration of multiple cell subsets, including T cells, eosinophils, monocytes, and NK cells, to sites of inflammation during autoimmune and inflammatory processes. This migration correlates with an observed increase in the development of diabetes.	[67,68,69]
CXCL14 (BRAK)	Adipose tissue, mast cells, healthy epithelial tissue such as intestines and kidneys, tumor cells, fibroblasts and macrophages	-	CXCL14 exhibits tumor inhibition, antibacterial, anti-inflammatory properties, and modulates cell migration.	[72,73,74]
CCL2 (MCP-1)	Tumor cells, monocytes, macrophages, endothelial cells, inflammatory cells	CCR2	CCL2 is a secreted factor expressed during normal immune surveillance, as well as in response to injury or infection. It influences a variety of tumors and diseases, including atherosclerosis, multiple sclerosis, and diabetes. Furthermore, CCL2 plays a critical role in the immune system by attracting immune cells to adipose tissue through chemotaxis, which is vital for macrophage infiltration into adipose tissue.	[10,80,81,82,83,84,85,86]
CCL3 (MIP-1α)	Macrophages, B cells, tumor cells and dendritic cells	CCR1, CCR5	CCL3 plays a crucial role in the pathophysiology of NAFLD by participating in the recruitment of neutrophils to sites of inflammation. Additionally, CCL3 contributes to cancer progression by binding to receptors that induce movement and infiltration of colorectal adenocarcinoma cells.	[14,90,91,92,93,94]
CCL5 (RANTS)	NK cells, T cells, dendritic cells, macrophages, and monocytes.	CCR1, CCR3 CCR5	CCL5 plays a role in physiological processes such as cell migration and immune response, and has a crucial role in anti-tumor activity.	[16,95,96,97]
CCL7 (MCP-3)	Tumor cells, fibroblasts, endothelial cells, vascular smooth muscle cells, and bone marrow mononuclear cells	CCR1, CCR2, CCR3, CCR5	CCL7 binds to specific receptors, promoting the migration and aggregation of monocytes and macrophages, and plays a role in immune and inflammatory responses as well as tumor development.	[99,100,101]
XCL1	CD8^+^ T cells, NK cells, mast cells, and activated Th1- polarized CD4^+^ T cells.	XCR1	XCL1 attracts activated T cells and NK cells, and its binding to XCR1 receptors may alleviate various inflammatory diseases. Enhancing XCL1-XCR1 signaling could improve treatments for severe infections and tumors, contributing to bactericidal activity and immune responses.	[105,106,107,108,109,110,111,112]

Note: IL-8: interleukin-8; IP-10: interferon-gamma-induced protein 10; BRAK: Breast and Kidney-Expressed Chemokine; MCP-1: monocyte chemotactic protein-1; MIP-1α: Macrophage Inflammatory Protein-1 alpha; RANTES: regulated upon activation, normal T cell expressed and secreted; MCP-3: monocyte chemoattractant protein-3; NK: natural killer cells; NAFLD: non-alcoholic fatty liver disease; MIG: monokine induced by interferon-γ.

**Table 2:** The role of different chemokines in obesity.

Chemokine Name	Primary Role	References
CCL2 (MCP-1)	High expression of CCL2 in obese tissues, which is closely associated with obesity-induced cardiovascular disease and insulin resistance, promotes the migration of monocytes to adipose tissue.	[10,11,12]
CCL5 (RANTES)	CCL5 is associated with obesity-induced cardiovascular disease and insulin resistance, and has effects on mitochondrial oxidative phosphorylation and fatty acid metabolic pathways. When the binding signal of CCL5 and its receptor is enhanced, it inhibits AMPK activity, which leads to a decrease in lipolysis and oxidative metabolism.	[16,17]
CXCL14 (BRAK)	CXCL14 levels are reduced in obese patients, with an inverse correlation with inflammation-related gene expression and a positive correlation with markers of insulin sensitivity.	[75]
CCL3 (MIP-1α)	CCL3 expression is elevated in the obese state and correlates with the pathogenesis of NAFLD.	[14,94]
CCL7 (MCP-3)	Overexpression of CCL7 protects the organism in a mouse model of diet-induced obesity and hepatic steatosis.	[102]
CXCL8 (IL-8)	CXCL8 plays a key role in the pathophysiology of obesity, driving liver inflammation and injury.	[59]
CXCL9 (MIG)	CXCL9 is involved in immune and inflammatory responses and plays an important role in tumor immunosurveillance and antitumor immunity.	[63,64,65,66]
CXCL10 (IP-10)	Elevated circulating levels of CXCL10 in obese patients are associated with obesity markers and may be involved in insulin resistance and obesity-related cardiovascular complications.	[67,68,70,71]

Note: IL-8: interleukin-8; IP-10: interferon-gamma-induced protein 10; AMPK: AMP-activated protein kinase; BRAK: Breast and Kidney-Expressed Chemokine; MCP-1: monocyte chemotactic protein-1; MIP-1α: Macrophage Inflammatory Protein-1 alpha; RANTES: regulated upon activation, normal T cell expressed and secreted; MCP-3: monocyte chemoattractant protein-3; NAFLD: non-alcoholic fatty liver disease; **MIG**: monokine induced by interferon-γ.

## 4. Role of Chemokines in Obesity

Obesity is associated with a chronic inflammatory state in which chemokines play an important role, influencing the recruitment and activation of immune cells. Chemokines and their receptors direct circulating leukocytes to the site of inflammation and injury [47]. For example, the CC and CXC chemokine families are primarily responsible for attracting monocytes and polymorphonuclear leukocytes to sites of chronic and acute inflammation, respectively. In addition, these chemokines are involved in the regulation of adaptive immune responses and play a key role in the pathogenesis of several diseases [42,45,46,47].

### 4.1. CC Critical Role of Chemokines in Obesity-Related Inflammation

In the state of obesity, adipose tissue releases various chemokines and inflammatory factors, including MCP-1, TNF-α, IL-1β, and IL-6. These factors can activate signaling pathways such as Nuclear Factor kappa B (NF-κB) and c-Jun N-terminal Kinase (JNK), and may lead to inflammatory responses. Concurrently, obesity may also change adipocyte size and number to impact chemokine and inflammatory factor expression and release [113]. Examples include the association between MCP-1 and RANTES and obesity [49,114].

This is accompanied by the release of a large number of chemokines. These chemokines not only affect diseases related to the immune system, but may also have potential significance in the treatment of obesity [49]. Chemokines CCL2 and CCL5 have been associated with elevated levels in adipose tissue in obesity [114]. Elevation of CCL2 in the adipose tissue of patients with obesity attracts monocytes, resulting in inflammation and insulin resistance within the adipose tissue. Conversely, CCL2 levels decline accompanied by weight loss. Additionally, CCL2’s interaction with proteoglycans promotes tissue inflammation and fibrosis [10]. The increased expression of free fatty acids in circulation may serve as a trigger for the upregulation of these chemokines through the inhibitor of κB kinase β (IKKβ) and JNK pathways. The resultant overproduction of inflammatory cytokines and chemokines further exacerbates the inflammatory response associated with obesity [11,115]. In terms of disease prevention, weight loss has been shown to reduce serum concentrations of the chemokine MCP-1 in obese men with metabolic syndrome and to improve markers associated with renal injury, suggesting that renal tubular injury can be ameliorated by reducing inflammation [116]. In addition, chemokines may also serve as a potential biomarker for predicting childhood obesity and insulin resistance [117].

During obesity, the expression and activity of inflammatory factors are altered, including the up-regulation of CCL2, which leads to more macrophages being recruited into adipose tissue and exacerbate metabolic inflammation [10,118]. Chemokines and inflammatory mediators are key regulators of the inflammatory responses and pathophysiological processes in obesity-related conditions [119]. In obesity, adipocytes secrete more adipokines that recruit and activate macrophages, and intensify inflammation within adipose tissue. While activated macrophages in adipose tissue secrete additional mediators to recruit more macrophages and perpetuate a positive feedback loop that sustains adipose tissue inflammation, this persistent inflammation contributes to the development of insulin resistance [120]. In a mouse model of diet-induced obesity and hepatic steatosis, overexpression of CCL7 protects the organism from these diseases [102]. Further studies have found that CCL7 and adiposity play an important role in promoting migration and infiltration of inflammatory cells [103]. In terms of obesity and DSPN, CCL7 is a potential biomarker of inflammation, reflecting its correlation with BMI and waist circumference. CCL7 may partially mediate the relationship between obesity and DSPN [104]. Therefore, the key role of CC chemokines in obesity-associated inflammation and metabolic disorders emphasizes their importance as potential targets for the treatment of obesity and related complications.

### 4.2. CXC Chemokines Modulate Obesity Inflammation and Metabolism

CXC chemokines regulate inflammation associated with obesity, impact immune cell infiltration, tumor progression, and insulin resistance. Specific factors such as CXCL16/CXCR6, CXCL9/10/11, and CXCL12/CXCR4 are implicated in cellular recruitment. The inhibition of these chemokines demonstrates therapeutic promise, yet further research is required to elucidate their molecular pathways [51]. Studies have demonstrated that adipocytes secrete the CXCL12 chemokine, which attracts macrophages into adipose tissue, inducing inflammatory responses and impairing insulin sensitivity. In murine models of obesity, both the expression and secretion of CXCL12 are elevated. Inhibiting the CXCL12 signaling pathway has been shown to effectively decrease macrophage accumulation in adipose tissue and aid in restoring insulin sensitivity. These findings reveal a potential regulatory role for CXCL12 in obesity-related metabolic disorders [121]. Another chemokine, CXCL14 regulates energy metabolism. CXCL14 counteracts metabolic disorders and insulin resistance associated with obesity by stimulating brown adipose tissue activity, promoting browning of white adipose tissue, and recruiting M2-type macrophages [122]. A related study found that overexpression of CXCL1 in muscle attenuates fat accumulation induced by a high-fat diet, and enhances the muscle’s ability to oxidize fatty acids. This suggests the capacity of CXCL1 to prevent and treat obesity [123]. The regulatory role of CXC chemokines offers new strategies for obesity treatment, particularly in reducing inflammation and improving metabolic health.

## 5. Effect of Chemokine Knockout or Pharmacological Inhibition on Obesity

### 5.1. Effects and Mechanisms of Pharmacological Inhibition of Chemokines on Obesity

#### 5.1.1. Analysis of the Role and Effects of CXC Inhibitors in the Regulation of Inflammation and Metabolism in Obesity

Pharmacological inhibition of chemokines offers a novel approach to obesity treatment by modulating inflammatory state and metabolic pathways. Chemokine inhibitors have been studied and have been demonstrated to play an important role in the obesity process. Cui et al. [18] administered a CXCL8 analogue, CXCL8 (3–72) K11R/G31P (briefly named G31P), an inhibitor of CXCR1 and CXCR2, to db/db *mice* to evaluate its protective effects against immunometabolic disorders. The treatment significantly improved glycemic control, insulin sensitivity, and lipid metabolism by modulating the inflammatory response. G31P effectively reduced the infiltration of inflammatory cells and circulating cytokine levels in the liver and adipose tissues. Meanwhile, this treatment promoted the activation of anti-inflammatory M2 macrophages and attenuated obesity-induced inflammation. While CXC chemokine inhibitors such as G31P show a prospective power in regulating obesity-related metabolism. However, current research on CXC inhibitors leaves much to be desired.

#### 5.1.2. Effects of CC Inhibitors and Other Pharmacologic Interventions on Obesity and Metabolic Disorders

In addition to other antagonists, specific CCR2 antagonists have shown efficacy in reducing blood glucose and enhancing insulin sensitivity in obesity treatments. They also contribute to the attenuation of lipid accumulation and inflammation in both liver and adipose tissue. These antagonists regulate lipid metabolism by diminishing macrophage accumulation in adipose tissues. However, they have different effects in lipid levels. Some antagonists may reduce triglycerides, but they do not change total cholesterol consistently [124].

In addition, propagermanium, a chemokine receptor CCR2 antagonist, has demonstrated efficacy in mitigating metabolic disorders associated with a high-fat diet-induced obesity mouse model. It attenuates weight gain and visceral fat accumulation, decreases the aggregation of pro-inflammatory M1-type macrophages, and promotes polarization towards anti-inflammatory M2-type macrophages in adipose tissue. Propagermanium also enhances insulin sensitivity and modulates levels of lipocalin and leptin, as well as augments lipoxygenase activity in hepatic and muscle tissues. It reduces hepatic triglyceride levels and curbs inflammation. Furthermore, propagermanium seems to decrease leptin resistance in adipose tissue. All of this suggests its role in impeding obesity through multifaceted pathways [125].

Tamura et al. [126] investigated the effects of propagermanium (a CCR2 inhibitor) on obesity and type 2 diabetes by administering a standard diet supplemented with propagermanium to 6-week-old db/db *mice* for 12 weeks. The treatment with propagermanium significantly mitigated weight gain, visceral fat accumulation, and adipocyte hypertrophy. It also reduced macrophage infiltration and inflammatory responses in adipose tissue. Moreover, it enhanced glucose tolerance, insulin sensitivity, and decreased triacylglycerol levels in the liver. This was accompanied by a reduction in hepatic fat synthesis, TNF-α expression, and monocyte migration. These findings indicate that targeting adipose tissue inflammation via CCR2 inhibition can potentially alleviate obesity and its associated metabolic disturbances.

Cenicriviroc (CVC) is an orally administered dual inhibitor of the C-C chemokine receptors CCR2 and CCR5. It effectively hampers monocyte infiltration in the liver by curtailing the heightened expression of these chemokines in hepatic injury. Consequently, CVC administration leads to a reduction in serum cholesterol and triacylglycerol levels in *mice* with steatohepatitis. This results in less weight gain and fat accumulation in the liver. Furthermore, it ameliorates insulin resistance and lowers hepatic triglyceride content [127].

The administration of a neutralizing monoclonal antibody against CCL4 enhanced glucose metabolism and lipid profiles, altered the composition and functionality of the intestinal microbiota, mitigated inflammation, and aided in controlling insulin resistance and the progression of hyperglycemic symptoms in a mouse model of diet-induced diabetes [128]. Pharmacological inhibition of bile salt hydrolase (BSH) not only decelerates the advancement of obesity-associated colon cancer in a high-fat diet model but also exhibits antitumor effects by disrupting the β-catenin/CCL28 signaling pathway [129].

### 5.2. Effects and Mechanisms of Knockdown of Chemokines on Obesity

#### 5.2.1. Dual Effects of CXC Knockout on Obesity

Obese adipose tissue secretes chemokines that may have detrimental effects on the organism. *Mice* with MCP-1 gene knockouts showed reduced adipogenesis and less adipocyte hypertrophy when fed a high-fat diet compared to wild-type *mice*. These knockout *mice* also exhibited enhanced insulin sensitivity, decreased insulin secretion, reduced blood levels of inflammatory and angiogenic factors, and diminished incidence and growth of mammary tumors [130]. Conversely, some gene knockouts resulted in opposite effects. CXCR4 possesses multiple roles in adipocytes. Firstly, it regulates the infiltration of inflammatory cells into adipose tissue, thus diminishing obesity-associated inflammation. Secondly, CXCR4 is involved in the thermogenic response of brown adipose tissue to augment energy dissipation, which results in a decline in fat deposits and obesity. However, knockout of CXCLR4 may aggravate obesity and damage the thermogenic capacity of brown adipose tissue, along with an increase in inflammatory cells within white adipose tissue. Moreover, CXCR4 interacts with its ligand CXCL12, influencing cell migration and localization. CXCR4 is pivotal for embryonic development, as well as for the homeostasis and functioning of the immune and stem cell systems [131].

#### 5.2.2. Effects of CC Knockout on Obesity and Metabolic Disorders

Duffy antigen receptor for chemokines (DARC) is a multifunctional nonsignaling receptor and expresses on red blood cells and other cell types. It regulates inflammation and cell migration by binding to chemokines such as MCP-1. The absence of the DARC gene is associated with exacerbated insulin resistance and adipose tissue inflammation under a high-fat diet. Experiments have demonstrated that *mice* lacking the DARC gene exhibit an increase in body weight, reduced glucose tolerance, decreased insulin sensitivity, and enhanced inflammatory markers in adipose tissue [132].

Zhou et al. [133] reported that knocking out CCL5 in normal weight *mice* improved glucose tolerance and pancreatic islet function, whereas a knockout of CCL5 in obese *mice* exacerbates insulin resistance and hepatic fat accumulation. Additionally, this knockout may increase the expression of other chemokines to enhance immune responses. These findings illustrate the multifaceted effects of CCL5 knockout on metabolism in *mice*, but these effects may depend on the animal’s weight status. However, Chan et al. [16] demonstrated that *mice* deficient in CCL5 gained less weight and exhibited lower levels of blood glucose and insulin, as well as improved insulin sensitivity, when fed a high-fat diet compared to their wild-type counterparts, and these effects occurred independently of food intake. The absence of CCL5 also led to reduced lipid accumulation and inflammation in adipose tissue, facilitated a shift in macrophage polarization from the M1 to the M2 phenotype, and modulated the levels of pro-inflammatory and anti-inflammatory cytokines. Furthermore, CCL5 influenced the composition of immune cell populations within the adipose tissue, potentially contributing to a decreased risk of developing obesity-associated insulin resistance.

**Figure 2:**
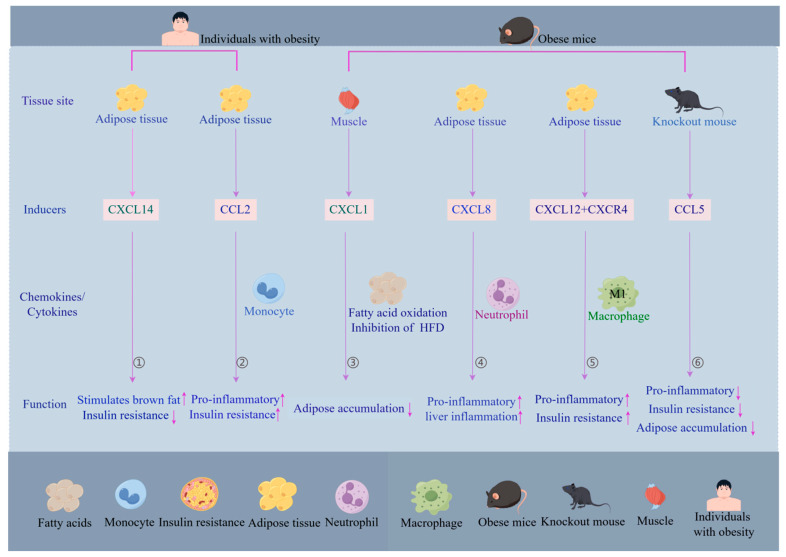
The role of chemokines in *human* and murine tissues and their impact on obesity-related inflammatory responses and insulin resistance (by Figdraw). ① Adipose tissue secretes chemokine (CXCL14), which activates brown adipose tissue and helps reduce insulin resistance. ② Heightened expression of CCL2 in the adipose tissue of obese individuals promotes the recruitment of monocytes to the tissue, leading to inflammation and subsequent insulin resistance. ③ Overexpression of CXCL1 in mouse muscle tissue reduces fat accumulation associated with a high-fat diet and enhances oxidative capacity for fatty acids in the muscles, which may indirectly contribute to an increase in muscle mass. ④ CXCL8 expression in adipose tissue mediates neutrophil recruitment, which may influence liver inflammation associated with obesity. ⑤ CXCL12, secreted by adipose tissue, interacts with its receptor CXCR4, which facilitates the recruitment and accumulation of M1 macrophages. This process is implicated in the exacerbation of the inflammatory response and the development of insulin resistance. ⑥ *Mice* with a systemic knockout of CCL5 exhibit attenuated inflammatory responses, reduced adiposity, and improved insulin sensitivity when fed a high-fat diet. The flow chart was drawn by Figdraw.

CCR2 is a chemokine receptor that is closely associated with obesity-induced kidney injury and its associated oxidative stress and endoplasmic reticulum stress. Knockdown of CCR2, even in the context of obesity, can attenuate renal injury, prevent excessive immune cell infiltration, and reduce inflammation and fibrosis, thus improving renal function [134]. Although pharmacological inhibition and knockout chemokines show potential in obesity treatment, further studies are needed to determine their long-term effects and safety, Figure 2.

## 6. The Effect of Exercise on Weight Management and the Role of Chemokines in This Process

### 6.1. Effectiveness of Exercise for Weight Management

With obesity increasing globally, exercise as an effective weight management method becomes more important. The main cause of obesity is an imbalance between energy intake and expenditure [135]. Fortunately, exercise can improve this imbalance, which means engaging in aerobic, resistance, and combined exercise to promote energy expenditure and weight loss.

#### 6.1.1. Aerobic Exercise

Aerobic workouts rely on oxygen for energy, which helps to boost cardiorespiratory fitness, cardiovascular health, improve metabolism, and promote weight loss. Types of aerobic exercise include walking, brisk walking, light jogging, and cycling [136,137]. Aerobic exercise optimizes adipose metabolism by increasing subcutaneous fat utilization, improving response to insulin, promoting mitochondrial activity, reducing inflammation and adipose tissue browning [138]. Single bouts of aerobic exercise are associated with an increase in M2-type macrophages and neutrophils in skeletal muscle, which corresponds to improved insulin sensitivity under obese conditions [139]. Long-term aerobic training modulates adipokine secretion, protects muscle mitochondria from high-fat dietary injury, improves metabolic efficiency, reduces visceral fat and improves waist circumference [140,141,142]. For example, 12 weeks of aerobic exercise reduced body weight, decreased hepatic fat and inflammation, inhibited the *TLR4* signaling pathway, and enhanced the activity of ApoA5 in *mice*, effectively ameliorating NASH induced by a high-fat diet [31]. Furthermore, studies confirmed that both HIIT and MICT are effective in increasing energy expenditure and contributing to weight loss [143].

#### 6.1.2. Resistance Movements

Resistance training imposes incremental loads to the body by self-body weight or exercise equipment to enhance muscle strength and size. This training not only promotes muscle development, but also contributes to cardiovascular fitness, prevents osteoporosis, and manages body weight [136,144,145,146]. In animal models, resistance training has been shown to improve insulin resistance and glucose tolerance, reduce adipocyte size, and activate insulin pathways in skeletal muscle [147]. In addition, resistance training reduces visceral fat, body weight, adiposity, and blood pressure while improving fat-free muscle mass and strength, decreasing body fat percentage and enhancing muscle-related fitness [148,149,150,151]. Resistance exercise also combats obesity and its complications by enhancing insulin signaling, reducing inflammatory responses, promoting favorable adjustments within the NADPH oxidase system, and improving the efficiency of insulin-mediated glucose clearance [152,153].

#### 6.1.3. Integrated Movement Forms

Interdisciplinary treatment combining aerobic exercise and resistance training is particularly effective in adolescent obesity. This exercise has demonstrated its benefits in reducing body fat and visceral fat, lowering low-density lipoprotein (LDL), and cholesterol [154]. Integrated exercise not only improves the endurance of the cardiovascular system, but also enhances muscle mobility, effectively reduces fat deposition, and improves glycemic control with reduced insulin resistance [155]. Compared to resistance exercise alone, combined exercise is more effective in preventing and controlling obesity, especially in populations with a higher body fat percentage [156]. Elderly obese individuals can maximize fitness and oxygen consumption with this combined exercise, which facilitates improvement in body weight and lean body mass [157]. Compared with resistance training alone, integrated exercise substantially increases maximal oxygen consumption and enhanced muscle strength in individuals with obesity by combining aerobic and resistance exercise [158]. Integrated exercise also helps to reduce body fat content, BMI, and adiposity in children with obesity, while increasing lean BMI significantly reducing cardiovascular risk and obesity-related inflammation [159]. It has been shown that combined exercise during dietary weight loss significantly slows the decline in bone mineral density (BMD) in individuals with obesity. All ages and body mass index categories of people with obesity benefit from the protective effects of exercise on bone mineral density [160,161,162]. Based on integrated exercise to promote energy expenditure and fat oxidation through multiple mechanisms, it has significant benefits for improving obesity and promoting health. Therefore, it is recommended as the preferred option for weight management to enhance weight loss.

### 6.2. Role and Mechanisms of Chemokines in Exercise Weight Loss

Exercise as an effective method of weight loss, such as aerobic exercise, not only reduces subcutaneous fat in obese individuals, but also prevents chronic inflammation of skeletal muscle and insulin resistance. Furthermore, exercise modulates the NF-κB signaling pathway to impact body composition positively and ameliorate cardiovascular disease risk factors. During exercise for weight loss, it also causes changes in the levels of chemokines in skeletal muscle, including CCL2, CCL7, and CXCL1. Given age and biorhythm considerations, a new perspective of intervention through physical activity is offered for metabolic disorders [139,158,163,164,165]. In addition, exercise-induced weight loss reduces the volume of white adipose tissue and ameliorates tissue hypoxia, which leads to reduced chemokine expression [166]. For example, exercise upregulates RANTES levels in the adipose tissue and serum of obese *mice*, affects the immune system, may lead to weight loss and alter immune status within adipose tissue [167]. Relatedly, improvements in metabolic biomarkers such as blood pressure and blood glucose tend to coincide with exercise-induced weight loss, which is associated with reductions in visceral adiposity and expression of chemokines such as MCP-1 [168]. Thus, chemokines play a key role in the obese state by directing and attracting immune cells into adipose tissue [169].

In the obese state, increased expression of MIP-1α/CCL3 in adipose tissue is positively correlated with elevated levels of TNF-α and CD163 [94]. In contrast, the infiltration of inflammatory macrophages and CD8^+^ T cells can be reduced by exercise, which decreases the expression of TNF-α and IL-6 in adipose tissue and slows down adipose-generated inflammation [170]. Relevant studies have shown that exercise reduces the expression of TNF-α and IL-6 in adipose tissue by decreasing the infiltration of inflammatory macrophages and CD8^+^ T cells. These cells are involved in adipocyte differentiation and fat metabolism, and thus exercise contributes to fat loss [31]. Recruitment of hepatic macrophages can be manipulated by knockdown of CCL3, altering the M1/M2 balance and thereby ameliorating steatohepatitis induced by a high-fat diet [14]. Downregulation of CCL3 also helps to reduce inflammatory cell infiltration in adipose tissue and decreases the expression levels of genes involved in fatty acid synthesis and fatty acid oxidation [171].

It was shown that ccr7^−/−^ *mice* in an obese mouse model were able to counteract high-fat diet-induced weight gain and insulin resistance by increasing calorie consumption and decreasing inflammatory activity. This reveals the regulatory role of the CCL19-CCR7 signaling pathway in the development of obesity and its associated state of insulin resistance [172]. In the development of obesity pathology, weight loss has been shown to effectively lower CCL2 levels, thereby alleviating inflammation and reducing the risk of related conditions, such as macrophage activation, hepatocellular carcinoma, rheumatoid arthritis, and multiple myeloma [10,86]. Targeted knockdown or pharmacological inhibition of these chemokines, such as the deletion of the CCR2 receptor that binds to the CCL2 ligand, has been shown to ameliorate metabolic abnormalities in obese individuals. This intervention can help to reduce fat accumulation, oxidative stress, and endoplasmic reticulum stress [134]. similarly, exercise reduces CCL2 secretion that also reduces the inflammatory response associated with obesity [173]. Further, knockdown of MCP-1 enhances M2 polarization in adipose tissue, promotes the conversion of white to brown fat, and increases the activity of brown adipose tissue [174].

**Figure 3:**
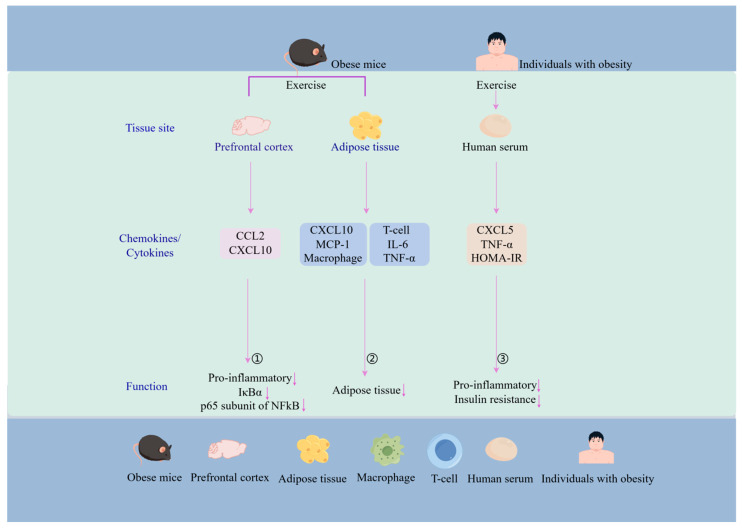
Exercise modulates chemokine expression in *human* and murine tissues to influence obesity-related inflammatory responses (by Figdraw). ① Exercise reduces obesity-induced inflammatory responses, including IκBα, p65/RelA, by decreasing CXCL10 and CCL2 expression in the prefrontal cortex of *mice*. ② Exercise downregulates the expression of CXCL10, CCL2, CD8^+^ T cells, TNF-α, and IL-6, which are associated with obesity and immunometabolic disorders in *mice*. This modulation may lead to a reduction in inflammatory responses and adiposity. ③ Exercise has been shown to reduce serum levels of CXCL5 and TNF-alpha in *humans*, as well as lowering the HOMA-IR index, which can help improve insulin sensitivity and reduce inflammatory conditions.

In obesity-associated immune surveillance, CXC chemokines have a critical impact on insulin resistance and lipid metabolism by recruiting immune cells through binding to receptors and modulating inflammation. Physical activity, as part of an integrated lifestyle, is effective in reducing levels of the chemokine IP10/CXCL10 in the immune system, thus potentially helping to alleviate obesity-related immunometabolic problems [51,175]. Altered gene expression of CXCL10 and CCL2 chemokines in prefrontal cortex was associated with exercise-induced weight loss. Exercise prevents the induction of these chemokines to reduce the inflammatory response associated with obesity. Although exercise has a smaller effect on the expression of inflammation-related genes in the prefrontal cortex, it overall reduces specific functional gene clusters associated with inflammation. This includes the expression of the p65 subunit of NFkB and IKBα [173]. A study reported a subsequent decrease in serum CXCL5, HOMA-IR, and TNFα levels in sedentary women with obesity after a 12-week exercise training program. These findings indicate that exercise-induced weight loss reduces serum CXCL5 concentrations and improves insulin resistance [176]. In addition, low-intensity exercise significantly affected the expression of B Lymphocyte Chemoattractant (BLC), eotaxin, Granulocyte Chemotactic Protein 2 (GCP-2), Monocyte Chemoattractant Protein 4 (MCP-4) and Neutrophil Activating Peptide 2 (NAP2) expression. These chemokines play an important role in mediating chemotactic responses [177]. The mechanisms by which exercise regulates the expression of these chemokines may provide a novel strategy for intervening in obesity, particularly in terms of reducing inflammation and improving metabolic health, Figure 3.

## 7. Conclusions

This paper provides insight into how exercise influences the pathogenesis of obesity by regulating chemokine expression. We revealed the remarkable effect of this intervention in reducing pro-inflammatory chemokines such as CCL2, CCL3, CCL5, CCL7, and CXCL8. It also highlights the role of physical activity in regulating complex immune factors like CXCL9, CXCL10, and XCL1, and in enhancing the expression of the protective chemokine CXCL14. These changes improve insulin sensitivity, promote healthful fat metabolism, decrease inflammatory cell infiltration, and enhance metabolic activity in adipose tissue, offering new research directions for the intervention and management of obesity and its complications. Future studies should explore in greater depth the specific mechanisms through which physical activities modulate individual chemokines and assess the optimal effects of different types and intensities of activities, such as aerobic, combined aerobic and resistance exercises, and comparing HIIT to MICT on chemokine expression, in obesity management. Particularly, this study directs attention towards personalized obesity intervention strategies.
